# Evaluating the use of whole genome sequencing for the investigation of a large mumps outbreak in Ontario, Canada

**DOI:** 10.1038/s41598-019-47740-1

**Published:** 2019-08-30

**Authors:** P. J. Stapleton, A. Eshaghi, C. Y. Seo, S. Wilson, T. Harris, S. L. Deeks, S. Bolotin, L. W. Goneau, J. B. Gubbay, S. N. Patel

**Affiliations:** 1 Laboratory Medicine & Pathobiology, University of Toronto, Toronto, ON Canada; 2 Public Health Ontario Laboratory, Public Health Ontario, Toronto, ON Canada; 3 Communicable Diseases, Emergency Preparedness and Response, Public Health Ontario, Toronto, ON Canada; 4 Dalla Lana School of Public Health, University of Toronto, Toronto, ON Canada; 5 Applied Immunisation Research and Evaluation, Public Health Ontario, Toronto, ON Canada

**Keywords:** Viral infection, Viral epidemiology

## Abstract

In 2017 Ontario experienced the largest mumps outbreak in the province in 8 years, at a time when multiple outbreaks were occurring across North America. Of 259 reported cases, 143 occurred in Toronto, primarily among young adults. Routine genotyping of the small hydrophobic gene indicated that the outbreak was due to mumps virus genotype G. We performed a retrospective study of whole genome sequencing of 26 mumps virus isolates from early in the outbreak, using a tiling amplicon method. Results indicated that two of the cases were genetically divergent, with the remaining 24 cases belonging to two major clades and one minor clade. Phylogeographic analysis confirmed circulation of virus from each clade between Toronto and other regions in Ontario. Comparison with other genotype G strains from North America suggested that the presence of co-circulating major clades may have been due to separate importation events from outbreaks in the United States. A transmission network analysis performed with the software program *TransPhylo* was compared with previously collected epidemiological data. The transmission tree correlated with known epidemiological links between nine patients and identified new potential clusters with no known epidemiological links.

## Introduction

Mumps virus is a single-stranded negative-sense RNA virus in the Rubulavirus genus of the *Paramyxoviridae* family, with a 15.3 kilobase (kb) genome that encodes 8 proteins. It is highly contagious and causes outbreaks of respiratory illness. Disease is normally self-limiting, but can be complicated by meningitis, encephalitis, orchitis or oopheritis^1^. While the incidence of mumps in developed countries has declined dramatically from the 1970s and onwards following the introduction of effective live-attenuated vaccines (https://www.canada.ca/en/public-health/services/immunization/vaccine-preventable-diseases/mumps/health-professionals.html), the past decade has seen a relative resurgence of mumps activity in some high income countries^2–4^. This increase has been attributed to waning of immunity in young adults who were immunized with one or two doses of measles, mumps and rubella (MMR) vaccine^5–7^.

In Ontario, mumps is a reportable disease. All cases are investigated, and the detection of a cluster of cases prompts outbreak investigation and control measures by public health authorities. Mumps outbreak investigations are labour intensive and require public health professionals to interview cases and identify transmission networks and potential common exposures in public settings; commonly these include post-secondary education settings, social gatherings or sporting events. Identifying links between individual cases is often challenging, as up to 40% of individuals with mumps are either asymptomatic, or present with primarily respiratory symptoms, and therefore lack the classic clinical presentation of parotitis^1^.

Epidemiological investigations can be complemented by molecular genotyping studies, which can help confirm or refute potential transmission events by comparing strain relatedness. The most widely used genotyping method for mumps virus involves sequencing a 316 nucleotide region of the small hydrophobic (SH) gene. This is usually the most variable region of the mumps genome and encodes a membrane associated protein whose function is incompletely understood. There are 12 distinct mumps genotypes, which are distributed globally. Most outbreaks in North America in recent years have been caused by Genotype G^8^. Genotyping using the SH gene is of limited utility during genotype G outbreaks, as the most variable region of this genotype is not the SH gene, but rather has been reported to be in non-coding regions of the genome^8,9^.

Whole genome sequencing (WGS) provides the ultimate resolution for genomic epidemiological investigations, by identifying single nucleotide variants (SNVs) between isolates. Even over the relatively short timeframe of a typical mumps outbreak (i.e. a few months), genome substitutions in RNA viruses are likely to arise frequently enough to allow sufficient discrimination of distinct lineages within the outbreak, and even individual transmission events. This approach has been described for outbreaks of other paramyxoviruses^10^. However, WGS of mumps was until recently performed infrequently, with only 110 full genome sequences available in the NCBI GenBank database (http://www.ncbi.nlm.nih.gov/Genbank/index.html) in July 2017 when this study commenced, compared with approximately 500 Zika virus genomes and over 2000 Zaire ebolavirus genomes.

In 2017, Ontario experienced a mumps virus outbreak which was the largest in the province since 2008. There were 259 cases reported (https://www.publichealthontario.ca/en/DataAndAnalytics/Pages/RDTO2016.aspx), 143 of which (55%) occurred in Toronto. Immunisation status was known for 155 Ontario cases (60%); of these 67 (43%) had received 2 or more doses of MMR vaccine. It was unclear from initial epidemiological investigations if the cases outside Toronto were part of the same outbreak, or represented a separate provincial cluster, potentially due to importation of cases from simultaneous outbreaks occurring elsewhere in North America. The most frequent common exposure for the Toronto cases was attendance at downtown bars (n = 70, 49%), and only 22 (15%) were linked with education settings^11^. This is in contrast to prior Ontario outbreaks, which were associated with secondary or post-secondary education settings^12^, and it therefore presented unique challenges for outbreak control efforts. Routine case finding and outbreak control measures were expanded to include targeted bar inspections to reinforce good infection prevention and control practices, and a social media campaign targeting young adults^11^.

Routine SH genotyping of isolates from 203 PCR confirmed cases indicated that 194 (96%) were genotype G. The limited resolution of SH genotyping could not help resolve transmission networks. We performed a retrospective study using WGS of a convenience sample of virus isolates from 27 cases from the first three months of the outbreak, 17 of them (63%) from Toronto. Our aims were to determine if the results of WGS and transmission network analysis correlated with epidemiological data, and to evaluate the feasibility and desirability of using this approach prospectively in future outbreaks.

## Results

### Phylogenetic analysis

**Figure 1:**
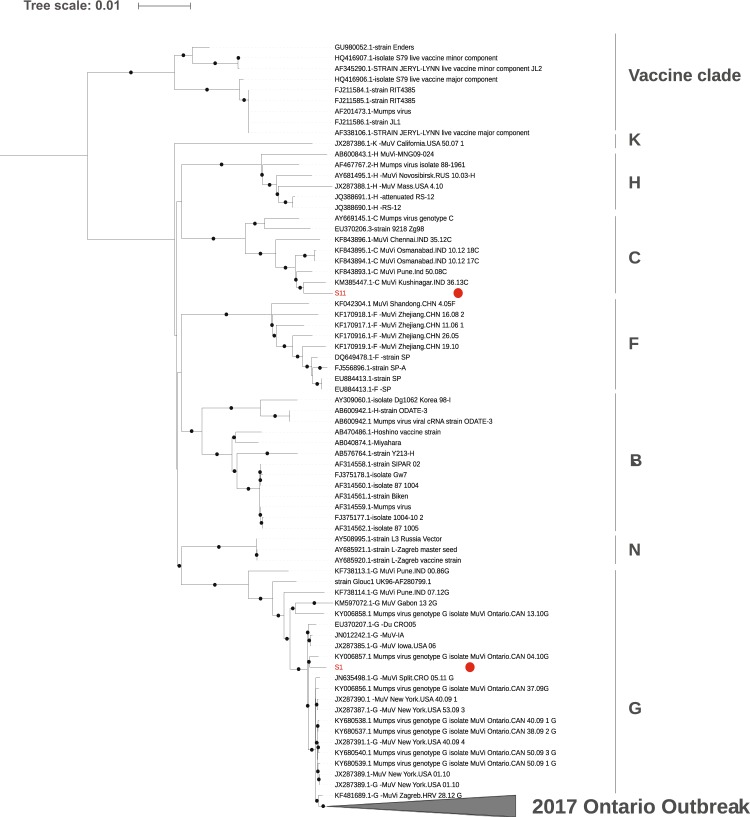
Phylogenetic tree created using whole genome alignment of 26 outbreak isolates and selected reference strains from NCBI GenBank. Text outside the tree indicates major phylogenetic groups. The main 2017 Ontario outbreak clade of 24 isolates is collapsed into a pyramid at the base of the tree. Two genomically distinct outliers from the outbreak, S1 (genotype G) and S11 (genotype C) are marked with red dots. The tree was created using the maximum likelihood method with iqtree v1.6.2 using the GTR + G model and 1000 ultrafast bootstrap approximations Black circles indicate nodes with >90% ultrafast bootstrap approximation support.

Amplicon based WGS was successful in 26 cases (96%). All 26 samples had average nucleotide coverage per site of at least 500X. Initial phylogenetic analysis revealed that isolates from patient 1 (S1) and patient 11 (S11) were genomically distinct from the main outbreak clade (Fig. 1).

Traditional SH genotyping differentiated S11, which is genotype C, from the main outbreak, but did not identify that case S1 was distinct from the other Genotype G strains and was therefore likely the result of an independent introduction of mumps virus. From the WGS phylogenetic tree it is apparent that S1 is more closely related to a sample we previously sequenced from a 2010 Ontario outbreak than it is to the other outbreak strains^13^.

Outbreak specific SNVs were identified by mapping reads of 23 isolates from the main outbreak clade against an in-group reference (S7). All samples had >99.9% site coverage compared to the reference assembly length of 15285 nucleotides. We identified 51 variable sites specific to the outbreak (Supplementary Dataset 1). All outbreak mutations were due to SNVs; there were no complex variants or insertions/deletions. The RNA-directed RNA polymerase L gene had the most variable sites (n = 23, 45%). There were only four variable sites in intergenic regions. A minority of SNVs (n = 14, 27%) were missense mutations that resulted in amino acid substitutions.

**Figure 2:**
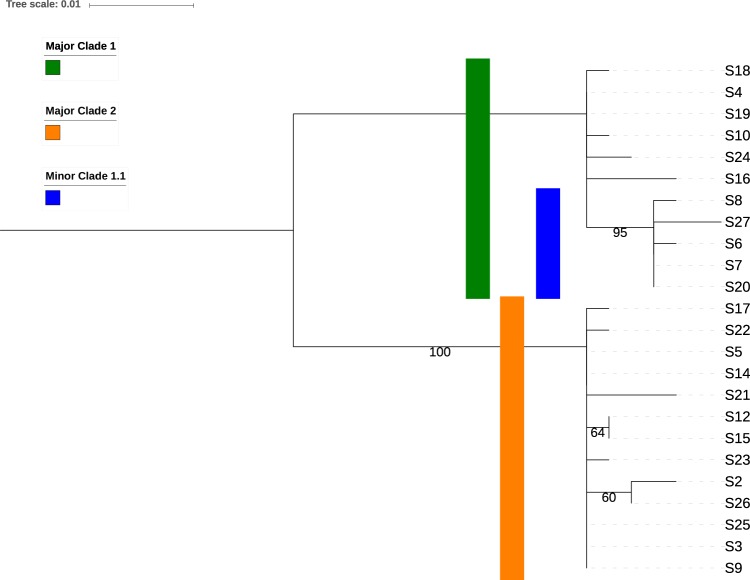
Phylogenetic tree of the outbreak clades. The two major and one minor clade are indicated with colored bars. The maximum likelihood tree was created by mapping reads to in-group reference (an S7 de-novo assembly). The tree is midpoint rooted and was constructed with iqtree using the GTR model with correction for ascertainment bias and 1000 traditional bootstrap replicates. Nodes with >50% bootstrap support are annotated with the support value.

Maximum likelihood (ML) tree analysis of the outbreak strains revealed two major clade and one minor clade (or cluster) with bootstrap support values >0.9 (Fig. 2). Interestingly, the minor clade consisted of five isolates that shared a common missense mutation in the SH gene (c.158C > T p.Ser53Phe), meaning that traditional SH genotyping can discriminate the minor clade from other samples, but does not identify the 2 major clades. The only other SNV in the SH gene (c.41T > C p.Ile14Thr, S2) was a phylogenetically uninformative singleton mutation. Overall, ML analysis helped us to identify clusters, but did not allow us to identify probable transmission events.

**Figure 3:**
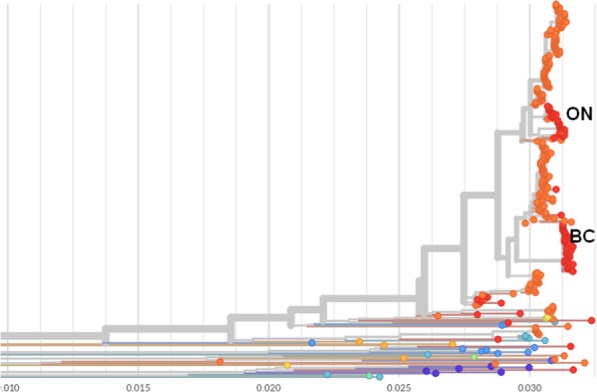
Phylogenetic tree of mumps isolates reproduced from nextstrain.org. Thick branches connect genotype G isolates. Orange circles indicate isolates from the USA and red circles isolates from Canada. The Y axis indicates percentage nucleotide diversity. ON, Ontario 2017 outbreak isolates; BC, British Columbia 2016 outbreak isolates.

Comparison of our strains with genotype G mumps virus circulating in North America was facilitated by the Nextstrain project (https://nextstrain.org/mumps/global), which includes sequences that are not publically available in GenBank. This analysis indicated our outbreak strains were more closely related to strains in the United States (US) than to an outbreak in British Columbia that occurred in summer 2016 (Fig. 3). Nextstrain analysis estimated the date of the most recent common ancestor for each of our major clades, using an ML discrete traits model. This indicated that sequences within each major Ontario clade coalesced in late 2016 or early 2017; the confidence interval (CI) for major clade 1 is 19^th^ September to 7^th^ January, and for major clade 2 is 1^st^ October to 12^th^ January. Both Ontario clades coalesced with clades from the US before they coalesced with each other in spring 2016.

When we examined the US association in greater detail, by ML comparison of our outbreak with 211 mumps genotype G strains from 2016/2017 outbreaks in the US, we identified 4 isolates which were closely related to our strains (Supplementary Fig. S1). Within Ontario major clade 1 is MuVs Massachusetts.USA 52.16 (GenBank accession MG986382), sequenced from a case with symptom onset on 27^th^ of December 2016, but no history of travel according to accompanying epidemiological metadata. Ontario major clade 2 is associated with MuVS Massachusetts.USA 13.16 (GenBank accession MF965213), with symptom onset on 31^st^ of March 2016 and a history of travel out of the country, but the specific destination of travel was not mentioned in the Massachusetts outbreak report^14^. Major clade 2 isolates are also closely related to 2 viruses from a Washington mumps virus outbreak in May 2017. This suggests that major clade 1 and 2 originated independently, from strains circulating in the US in 2016, with subsequent onward transmission from major clade 2 to Washington isolate MuVs Washington.USA 17.17. Only US 2016/2017 genotype G outbreak sequences were available for local comparison, so links to outbreaks in other countries cannot be definitively excluded.

### Phylogeographic analysis

**Figure 4:**
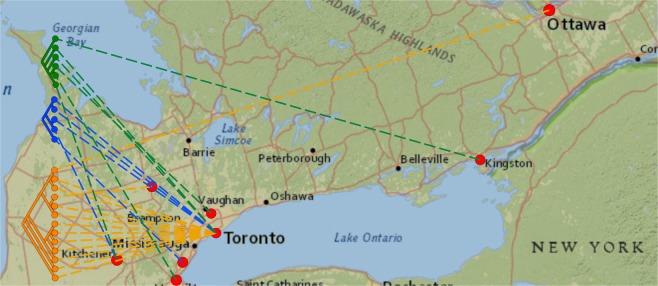
The outbreak phylogenetic tree superimposed on a map of Southern Ontario using the software program GenGIS v2.5.3. Red circles indicate the public health unit regions (PHUR) and are connected by colored dashed lines to the tree tips. The two major and one minor clades contain isolates from Toronto and from outlying PHURs, but major clade 2 is predominately associated with Toronto (11 cases, 85%). Tree tips are colored as follows: Green, isolates in major clade 1 (excluding those that are also contained in minor clade 1.1); Blue, isolates in minor clade 1.1; Orange; isolates in major clade 2. Map image is the intellectual property of Esri and is used herin under license. Copyright © 2018 Esri and its licensors. All rights reserved. Map sources: National Geographic, Esri, Garmin, HERE, UNEP-WCMC, USGS, NASA, ESA, Increment P Corp.

We examined the data for evidence to support an early hypothesis that distinct transmission networks existed in Toronto and other regions. We superimposed the main outbreak phylogenetic tree on a map of Southern Ontario to illustrate the geographic structure of the outbreak (Fig. 4). Most cases in major clade 2 (n = 11, 85%) are from the city of Toronto. However cases from major clade 1 and minor clade 1.1 come from both Toronto and surrounding regions. This indicates that the geographical structure of the outbreak was more complex than assumed, with transmission networks extending across the province, rather than forming distinct Toronto and outlying area outbreaks.

### Bayesian phylogenetic and transmission analysis

**Figure 5:**
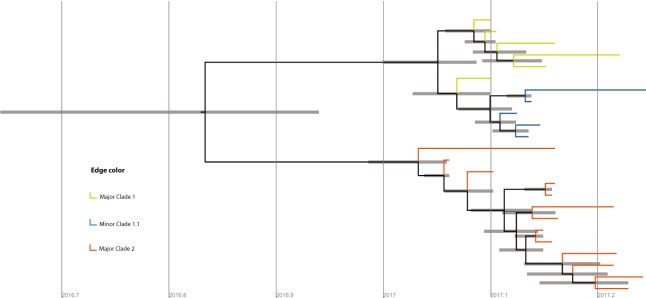
Timed tree from BEAST2 analysis. Time in decimal format is plotted on the Y axis. Nodes represent the mean estimate of time to most recent common ancestor (tMRCA) of descendant tips. Horizontal thick bars indicate 95% high probability distribution for the node height estimate. Tree tips are colored as follows: Green, isolates in major clade 1 (excluding those that are also contained in minor clade 1.1); Blue, isolates in minor clade 1.1; Orange isolates in major clade 2.

Bayesian phylogenetic analysis indicated that the time to Most Recent Common Ancestor (tMRCA) of our strains, and therefore the most likely date for origin of the outbreak, was October 25^th^ 2016, but with a 95% high probability distribution (HPD) for the date of August 23^rd^ to December 10^th^ 2016 (Fig. 5). This is earlier than the date of the first case detected public health units, January 9^th^. The differences in estimates for tMRCA from Nextstrain (spring 2016) and Bayesian analysis are likely due to the different models used, and the greater diversity of samples in the Nextstrain dataset. Although the Bayesian model is a better fit than the Nextstrain model for rapid outbreak growth dynamics, the Nextstrain model has the advantage of using other strains to refine the date estimates. Results of Nextstrain, ML and Bayesian analyses support the hypothesis that the two major clades were introduced by separate importation events in late 2016 or early 2017. The tMRCA estimates allow for the possibility that there was some degree of silent virus transmission after each importation (e.g. due to mumps infections that were asymptomatic or caused only non-specific respiratory symptoms) for several weeks, but are equally consistent with rapid case detection.

The mean molecular clock estimate for the mumps outbreak from Bayesian analysis was 2.24 × 10^−3^ substitutions/site/year (95% HPD 1.39, 3.1). This is higher than earlier mean clock estimates for mumps based on analysis of F-SH-HN gene sequence of 0.25 (0, 0.43), or 0.65 (0, 1.4) for synonymous sites only^15^. In contrast to the dataset on which the earlier estimate is based, our data incorporated numerous substitutions in the most variable region of our strains, the L polymerase gene, and consists of samples collected over weeks rather than decades. Viral molecular clock rates will be overestimated by up to several orders of magnitude when derived from samples collected over a short period, compared to samples taken over an evolutionary timescale where purifying selection plays a significant role^16^. As the transmission tree software *Transphylo* requires a timed tree containing only outbreak sequences, we accepted the higher clock estimate imposed by these limitations.

**Figure 6:**
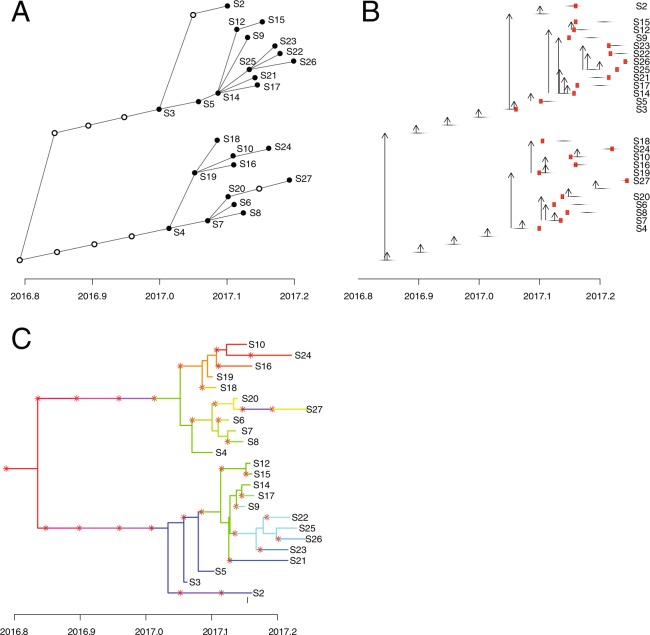
(**A**) Consensus TransPhylo transmission tree, plotted in ‘economical format’. The horizontal axis represents time in years, in decimal format. Filled circles represented sequenced cases. Empty circles represent putative unsampled cases, which the program has determined are required to reconcile the input phylogenetic tree with the pre-specified parameters for incubation period and infectious period, which must be provided as ranges for generation time and sampling time. If TransPhylo has determined that a direct transmission event is likely, then filled circles (sampled cases) are directly connected by a line. If sampled cases are separated by an unfilled circle (e.g. between S20 and S27), the program has determined that although the genomic sequences are similar, direct transmission is unlikely given known incubation and infectious period for mumps, and an unsampled intermediate case is required for transmission. (**B**) The transmission tree plotted in ‘standard’ format. Horizontal lines represent the infectious period for each sampled and unsampled case. Vertical arrows represent transmission from one case to another. Red squares indicate the time of patient sampling. Some of the purported transmission events are seen to reflect extreme assumptions e.g. S19 transmits to S18 at the earliest limit of the infectious period, and S18 is sampled before onset of symptoms. (**C**) The ‘colored phylogenetic tree’ view, consisting primarily of the timed tree also displayed in Fig. 5. The tree topology is unaltered, but Transphylo has colored the branches so that each case is represented by a unique color, and has inserted red asterisks to indicate each transmission event. This information provided by this view is the same as in panels A and B, and of the 3 potential Transphylo visual output formats, it is the least intuitive. Transphylo will maintain the clustering of cases seen on the input timed tree in its final outputs. Where 2 genomically similar cases share the same branch of the timed tree, but are separated by significant horizontal distance (S20 and S27, sampled weeks apart), it has colored a segment of the line purple, with red asterisks at the borders, to represent the unsampled intermediate case.

The timed tree generated by Bayesian phylogenetic analysis was input to *TransPhylo* to generate consensus transmission trees (Fig. 6). We found the ‘economical’ and ‘standard’ transmission tree viewing formats (Figs. 6A,B) easiest to interpret than the ‘colored phylogenetic tree’ format (Fig. 6C) when correlating the transmission tree with known epidemiological links. Epidemiologic data collected during routine public health investigation pertaining to 25 of the cases in our dataset were analysed separately to the genomic analysis, and identified three clusters of patients who shared common exposures; these clusters were then correlated with the transmission tree (Table 1).

**Table 1:** Correlation of epidemiological data for three clusters with *Transphylo* transmission tree.

Cluster	Cases	Epidemiological link	Transmission tree	Comments
A	S14, S17	Cases attended the same workplace and had collection of oral swab samples within one week of each other. No other case was identified at the workplace.	S14 transmits infection to S17	As the minimum mumps incubation period is 15 days, it is unlikely that S14 could have transmitted infection directly to S17
B	S4, S19, S10	S4 and S19 acquired their infection on the same date from a common source at a private residence. S10 lived nearby. Samples were collected from S4 and S19 on the same date and from S10 two weeks later	S4 transmits infection to S19 who transmits to S10 (and others)	*TransPhylo* model inferred that S4 was sampled after the infectious period, and S19 sampled at the beginning of the infectious period, to reconcile the fact that the collection date was the same for these samples but the timed tree suggested direct transmission from one to other
C	S12, S22, S23, S26 and S27	Cases linked by common exposure “Alcohol Serving Establishment (Bar/Tavern/Other)” and deemed to represent a potential cluster of cases associated with this general exposure setting.	S25^a^ transmitted to S22, S23 and S26. S14^b^ transmitted to S12 and S25. S27 is not linked to this cluster	S27 mumps virus strain is distinct from this cluster (it is part of a different major clade on phylogenetic analysis), so it can be confidently excluded from cluster C, despite the common exposure.

^a^No epidemiological data is available for this case. ^b^No recorded exposure to a Toronto bar.

Although most cases were not part of a cluster (no common exposures, n = 14, 58%), we identified three clusters (A - C) comprised of two, three and five cases respectively. The transmission tree proposes close links between the cases concerned. The only exception is case S27 from cluster C, where there is strong phylogenetic evidence to refute the proposed epidemiological link. In clusters A and B we observed discordance between the epidemiology and the transmission tree regarding the likely direction of transmission. In these clusters, *TransPhylo* analysis proposed direct transmission between patients, when the epidemiological data suggests they are more likely to have acquired the infection from a common source. The transmission tree identifies new purported links between cases that were not part of an epidemiological cluster previously (S7 transmitting to S6, S8 and S20), or that are now implicated as additional cases in an existing cluster (S16 and S18 are linked with the cases in cluster B). Due to the retrospective nature of this study, we were unable to perform further epidemiological investigations to support or refute these new links.

## Discussion

Genomic epidemiology based on sequencing an entire microbial genome is now routinely used for outbreaks of high profile pathogens such as Ebola virus and Zika virus^17,18^ and surveillance of pathogens such as *Mycobacterium tuberculosis*, where public health agencies expend enormous effort in identifying contacts who require chemoprophylaxis or treatment^19^. There are few studies published to date on the application of genomic epidemiology to mumps virus outbreaks; at the time the Ontario outbreak commenced we could find no studies correlating WGS results with outbreak investigations and to date only one study has been reported^14^.

In this retrospective study we developed protocols for amplicon sequencing from virus cultures and for identifying SNVs and clusters of closely related strains. Meaningful interpretation of clusters then requires close collaboration with public health professionals, who have specialist expertise in outbreak control, but who may not be accustomed to interpreting traditional visualisations of comparative genomic analyses, such as phylogenetic trees. We particularly focused on generating intuitive visual summaries of the phylogenomic data, such as maps illustrating the geographic structure of the outbreak, and transmission trees showing patient to patient spread.

We refined our previously published method for amplifying mumps virus DNA from virus culture samples^13^. At PHO, virus culture is routinely performed on all specimens that are reactive by RT-PCR, to assist with SH genotyping, and we have previously found the sensitivity of culture and RT-PCR to be broadly equivalent. Both virus culture and amplicon sequencing enrich for reads from the target virus rather than from the host or commensal bacteria. This allowed us to sequence our samples in a multiplex fashion on the Illumina MiSeq at PHO laboratories as part of runs where two thirds of sequencing capacity was allocated to bacterial pathogens for routine surveillance activities, and still achieve high depth of coverage of the target virus.

In future outbreaks, it may be desirable to sequence mumps virus directly from primary clinical specimens. This would reduce turnaround time by eliminating the culture step, which at our institution typically takes 7–10 days, but for samples weakly positive by RT-PCR may take up to 17 days or may fail to grow. Direct specimen sequencing would eliminate the possibility that SNVs could arise during cell culture passage. The impact of this potential confounder is unknown, as to our knowledge comparison of mumps virus sequence from before and after cell culture has not been performed. We investigated if it was possible to use our 9 amplicon protocol to sequence virus directly from 7 oral swabs positive for mumps virus, all with Ct values less than 33. However we could not obtain amplicon adequate product for all fragments under standard thermocycler conditions. Quick *et al*. recently described modified primer design and RT-PCR protocols for amplicon sequencing of Zika virus and chikungunya virus directly from clinical specimens^20^. Alternative methods for direct specimen sequencing include metagenomic approaches, either unbiased, or enriched with viral hybrid capture^21^. Hybrid capture has recently been shown to reliably recover sequence from buccal swabs positive by RT-PCR for mumps virus if the Ct value is under 30^14^. However in our experience the bioinformatics analysis was the time-limiting step, particularly optimisation of the models for generating timed trees and transmission trees.

Standard phylogenetic analysis based on SNV identification and construction of the ML tree was able to differentiate 24 outbreak strains from 2 non-outbreak strains. Only one Genotype C strain would have been differentiated using SH genotyping alone. The higher resolution provided by WGS allowed us to identify 2 major clade and 1 minor subclade within the outbreak. Identifying subclades is a starting point for identifying transmission events, since each clade is presumed to share a common ancestor, and therefore an epidemiological link. The phylogenetic tree was also used as input for a GenGIS phylogeographic analysis, which showed that strains from Toronto and surrounding regions were closely related. This was a question early in the outbreak, when the extent of strain sharing between public health units was unclear. Identifying strains shared between multiple health units may in the future help with multi-jurisdictional co-ordination of outbreak control efforts.

The transmission tree generated using *TransPhylo* was, in our opinion, superior to other means of visualising genomic data such as ML trees. Unlike ML trees it was able to incorporate available data about the timing of sample collection, which is a key factor to consider when using genomic data to identify transmission clusters^22^. *TransPhylo* used partial sampling data to generate a representation of case to case transmission, with clear illustrations of the assumptions the model made for each case to reconcile input data (a timed tree and dates of case sampling) with the constraints we imposed regarding the ranges for the infectious period and from virus acquisition to sampling. When we compared cases from 3 clusters with known epidemiological associations to the transmission tree, we found that the transmission tree independently identified close links between the cases. In the only exception, the results strongly support the genomic data over the epidemiologic data, since the case was infected with a strain from a different clade to other cases with the same category of epidemiological exposure.

Clearly *TransPhylo* has limitations; in 2 clusters it postulated direct patient to patient transmission when it is much more likely that cases acquired their infection from a common source. When we explored the inferences made by the model leading to errors for cluster B, we found the model made extreme assumptions about the time from case acquisition to sampling for a pair of cases (very early and very late in the course of illness respectively). This appears to have been done in order to reconcile an input timed tree showing considerable genomic distance between 2 strains, with the fact that the sampling date was the same for both cases. Although the transmission tree appears to be only a rough approximation of the true transmission network, we believe it may prove useful in future outbreaks. A key factor in future outbreak investigations will be the ability to generate accurate ML and timed trees from epidemiologically targeted or comprehensive case sequencing, rather than retrospective convenience sampling as in this study. The accuracy of phylogeographic and transmission models is constrained by the accuracy of the phylogenetic tree used as input, and the transmission model presented here would benefit from further refinement with independent datasets.

Whether mumps virus outbreaks have sufficient public health impact to warrant the expenditure of time and resources required to perform WGS based transmission network analysis is a subject for debate. Over the course of our outbreak investigation only a minority of cases had clear epidemiological links to other cases, so there is a need for genomic analyses to generate hypotheses with respect to transmission networks, and to inform additional case tracking measures. In most mumps outbreaks, interventions are limited to vaccination clinics for at risk populations, and to case isolation, as well as general messaging aimed at limiting transmission (i.e. advice to avoid sharing utensils and water bottles). These interventions were applied during the period of increased mumps activity in Ontario, but advice on immunisation was disseminated primarily through both traditional and social media, asking individuals to speak with their personal healthcare professional about immunisation. Prospective phylogenomic transmission network analysis could play an important role, by helping to identify hotspots for transmission and to define more precisely and vaccinate the population at risk. Public health agencies interested in prospectively applying these novel techniques should consider undertaking preparatory work to develop the necessary sequencing, bioinformatics and data visualisation methods. We are not aware of any study that has demonstrated a real, rather than potential, public health benefit from mumps WGS analysis, and to do so will require methods optimised in advance to deliver rapid results. Our study demonstrates that WGS of mumps virus is readily performed from virus culture and that traditional phylogenomic analyses are complemented by phylogeographic and transmission network analyses. Comparison of outbreak strains with sequences from traditional and novel data repositories helps identify potential international transmission events, which can then be correlated with results of epidemiological investigations. Transmission network analysis based on sequences from a small fraction of total cases generated results that were partly supported by known epidemiological associations. Limitations of our study included the small number of isolates sequenced, that our model inferred direct patient to patient transmission when acquisition from common sources was more likely, and that we were unable to further investigate potential new transmission links to confirm or refute them with epidemiological data. We believe that prospective phylogenomic analyses are needed to determine if WGS can be used to identify cryptic transmission chains in real-time and define the at-risk populations who would benefit from mumps containing vaccine.

## Materials and Methods

### Strain collection

Throat and buccal samples from all potential cases, identified either by primary clinicians or as a result of public health investigations of mumps, are routinely sent to the Public Health Ontario (PHO) Laboratories for analysis. All swabs were tested at PHO by reverse-transcription polymerase chain reaction (RT-PCR) targeting both SH and Fusion (F) genes, using an in-house assay adapted from protocols developed by Canada’s National Microbiology Laboratory (NML) and the US Centers for Disease Control and Prevention (CDC)^23,24^. Samples that were reactive in this assay were cultured in rhesus monkey kidney cell primary cell lines to assist with genotyping (Quidel Corporation, San Diego, CA). Total culture time is 17 days, including one passage at 10 days, although in our center most samples with Ct values < 30 are usually culture positive by 7 to 10 days. As this was a pilot study, we selected a convenience sample of 27 positive cultures, 17 of which were from Toronto, for sequencing. The first 20 cultures were selected randomly from samples collected in the first 2 months of 2017, at the beginning of the outbreak, and were sequenced in March. The remaining 7 cultures were selected from samples collected in the final 2 weeks of March and sequenced in June. We chose samples over a 3 month window to ensure adequate temporal signal in our dataset to enable us to perform phylogenetic molecular clock analysis.

### RNA extraction and sequencing protocol

Nucleic acid extraction and sequencing was carried out using a modified version of a tiling amplicon-based method to enrich the culture supernatant for viral RNA, which we previously used to sequence mumps virus from a 2010 Ontario outbreak^13^. We extracted RNA using either the QIAamp Viral RNA Mini Kit (Qiagen, Mississauga, ON) or the NucliSENS easyMAG instrument. For the initial eight samples we performed amplification of 18 overlapping amplicons, of mean length 977 bp. We optimised the protocol to reduce the number of amplicons, so for the last 19 samples we sequenced 9 amplicons of mean length 1958 bp (Supplementary Dataset 2 for primers). Amplification of the fragments in 96 well plates was performed on a SimpliAmp thermal cycler using the superscript III One Step RT-PCR system (Invitrogen,Thermo Fisher Scientific).

Amplicon fragments from individual samples were pooled together in equal amounts and cDNA concentration checked using a Qubit fluorometer. Mumps cDNA libraries were prepared with the Nextera XT kit. We checked the quality of the indexed libraries by Bioanalyzer. Sequencing on the Illumina MiSeq instrument was performed with V2 reagent kit (2 × 150 bp, Illumina Inc. San Diego, California, USA), according to the manufacturer’s instructions.

### Phylogenetic analysis

We removed adapters, primer sequences and low quality reads with Trimmomatic^25^. We created de-novo assemblies for each isolate using Spades v3.12.0^26^ as implemented in shovill v.0.9.0 (https: https://github.com/tseemann/shovill). Assembly errors were corrected by mapping trimmed reads back to each assembly with snippy v3.2-dev (https://github.com/tseemann/snippy).

We used MEGA7 to manually align our assemblies with a reference genome (accession JX287389) and with representative sequences of various mumps genotypes obtained from NCBI Genbank. We created a maximum likelihood (ML) tree from the full alignment using iqtree v1.6. To identify outbreak specific SNVs, we used snippy with default parameters to map sequencing reads of outbreak strains against an in-group reference (annotated de-novo assembly S7) which had >99% average nucleotide identify to other genotype G sequences in GenBank.

In order to compare our genotype G strains with as many whole genome sequences as possible, in addition to searching NCBI GenBank, we also conducted internet searches for sequences located outside of traditional data repositories. We retrieved 121 relevant Massachusetts outbreak sequences and associated clinical metadata from the bioRxiv preprint server for biology; an alignment of “clade-II” sequences was published as a supplement^14^. From github.com we retrieved 72 sequences from a research laboratory repository of sequences relating to a Washington outbreak, after obtaining permission from the researchers. Ultimately we created an ML tree from an alignment of 25 Ontario and 211 USA genotype G complete sequences from outbreaks occurring in 2016 and 2017, but we did not find sequences any from other countries in this period. Trees were visualised and prepared for publication using iTOL (http://itol.embl.de). We uploaded our outbreak clade genomes to the Nextstrain project (https://nextstrain.org).

### Phylogeographic analysis

To illustrate the phylogeographic structure of the outbreak we used the program GenGIS v2.5.3^27^ to combine the outbreak clade ML tree with a digital map with a location of the health unit where the sample was collected.

### Bayesian phylogenetic analysis

We required a time labelled phylogenetic tree as a starting point for the *TransPhylo* analysis, so we performed a Bayesian phylogenetic analysis of the outbreak strains using BEAST2 v2.4.7^28^. The complete consensus genome alignment of the outbreak clade was labelled with the collection date for each specimen. We assessed regression of root-to-tip distance in TempEst v1.5 and confirmed adequate temporal signal to proceed to Bayesian analysis. We used the birth-death skyline serial model as implemented in the BDSKY package v1.3.3, as an appropriate model for a RNA virus outbreak with changing dynamics due to the presence of resistant individuals and the depletion of the susceptible individuals^29^. We used the following parameters: HKY Model of evolution with empirical frequencies, gamma category count 4, proportion invariant sites 0.98 and a strict molecular clock. A strict clock was chosen as appropriate to a single outbreak in one location and was supported by the root-to-tip regression. When we attempted to run the analysis with a relaxed molecular clock we did not achieve convergence of the chain. We chose diffuse priors for the virus evolution rate, proportion of outbreak sampled, the rate at which patients become uninfectious and the reproductive number. The analysis was run for 40 million Markov Chain Monte Carlo (MCMC) simulations, with sampling from the posterior distribution every 4000 steps. Evaluation of the posterior probability of the parameters with Tracer v1.6 indicated adequate mixing of the chain, and all parameters achieved an effective sampling size (ESS) >200. The posterior sample of phylogenetic timed trees was summarised with TreeAnnotator v2.4.7, with the first 10% discarded as burn-in and an output tree of maximum sum of clade credibility with median node heights, which was visualised with Icytree^30^.

### *TransPhylo* analysis

For input to *TransPhylo* we used the time labelled phylogenetic tree along with initial estimates for the following parameters: sampling proportion Pi 0.1, date sampling of the outbreak stopped (last specimen collection, 2017.246) and a gamma distribution specifying the generation time, or the time between an individual’s primary infection and a secondary infection that they give rise to. Authorities such as WHO, CDC and the public health agency of Canada (http://www.health.gov.on.ca/en/pro/programs/publichealth/oph_standards/docs/mumps_chapter.pdf) give slightly different intervals and ranges for the incubation period and the period of infectiousness^31^. We specified a gamma distribution for the generation time with shape 64 and scale 0.000856, resulting in a mean of 20 days and 95% distribution of 14–30 days, in an attempt to incorporate WHO guidance on the incubation period (14–28 days, mean interval 16–18 days), and infectious period (−2 to +7 days from symptom onset), in a single distribution. We also specified a similar gamma distribution for sampling time (incubation time plus time from symptom onset to collection date) with a mean of 23 days (ws.shape 7, ws.scale 0.000856), specifying that our samples were most likely collected between 1 and 7 days from the onset of symptoms. This was an empiric estimate of time from symptoms to sampling, but subsequently our data linkage revealed that 23 of 25 cases with known onset and collection dates were within this time window (median 2 days, outliers were 0 days and 13 days).

*TransPhylo* uses MCMC simulation to analyse many thousands of possible transmission trees. Our simulation was run for 100000 MCMC simulations, with sampling of a tree every 1000 steps. We generated a consensus transmission tree from the output, with burn-in proportion of 0.5 and a minimum probability for inclusion of a partition in the consensus of 0.5.

### Epidemiological data

We compared the resulting transmission tree with previously collected epidemiological data recorded in the integrated Public Health Information System (*iPHIS*), which is Ontario’s electronic reporting system for reportable diseases. Of the 26 cases that were sequenced, 25 were matched to cases in iPHIS; one case could not be linked as the individual resided outside Ontario. Epidemiological data were extracted from iPHIS on April 20, 2018. PHO identified possible transmission clusters from the epidemiological data before reviewing the results of the genomic analysis. Clusters were defined as cases that had close contact with each other or that shared common exposures, as recorded in iPHIS.

### Ethical approval

The study protocol was approved by the PHO Ethics Review Board (ERB, File number 2017-053.01) and Privacy Office (Privacy assessment RRB-18-010). The ERB waived requirement for informed consent as the study satisfied the conditions of article 5.5A of the Tri-Council Policy Statement on Ethical Conduct for Research Involving Humans (TCPS2).

## Supplementary information

Supplementary Figure 1

Supplementary Dataset 1

Supplementary Dataset 2

Supplementary Dataset 3

## Data Availability

Complete genome sequences for the 26 strains have been deposited in NCBI GenBank with accession numbers MK033747 to MK033772. GenBank accession numbers and WHO names for each sequence are provided in Supplementary Dataset 3.
