# Supplementary material for: Evaluating the use of whole genome sequencing for the investigation of a large mumps outbreak in Ontario, Canada
Source: Sci Rep. 2019 Aug 30;9:12615. doi: 10.1038/s41598-019-47740-1 (PMC6717193; doi:10.1038/s41598-019-47740-1)
Supplement: Supplementary file 1 — Supplementary Figure 1 [file 41598_2019_47740_MOESM1_ESM.pdf]

Evaluating the use of whole genome sequencing for the investigation of a large mumps outbreak in Ontario, Canada

Stapleton PJ, Eshaghi A, Seo CY, Wilson S, Harris T, Deeks SL, Bolotin S, Goneau LW, Gubbay JB,Patel SN

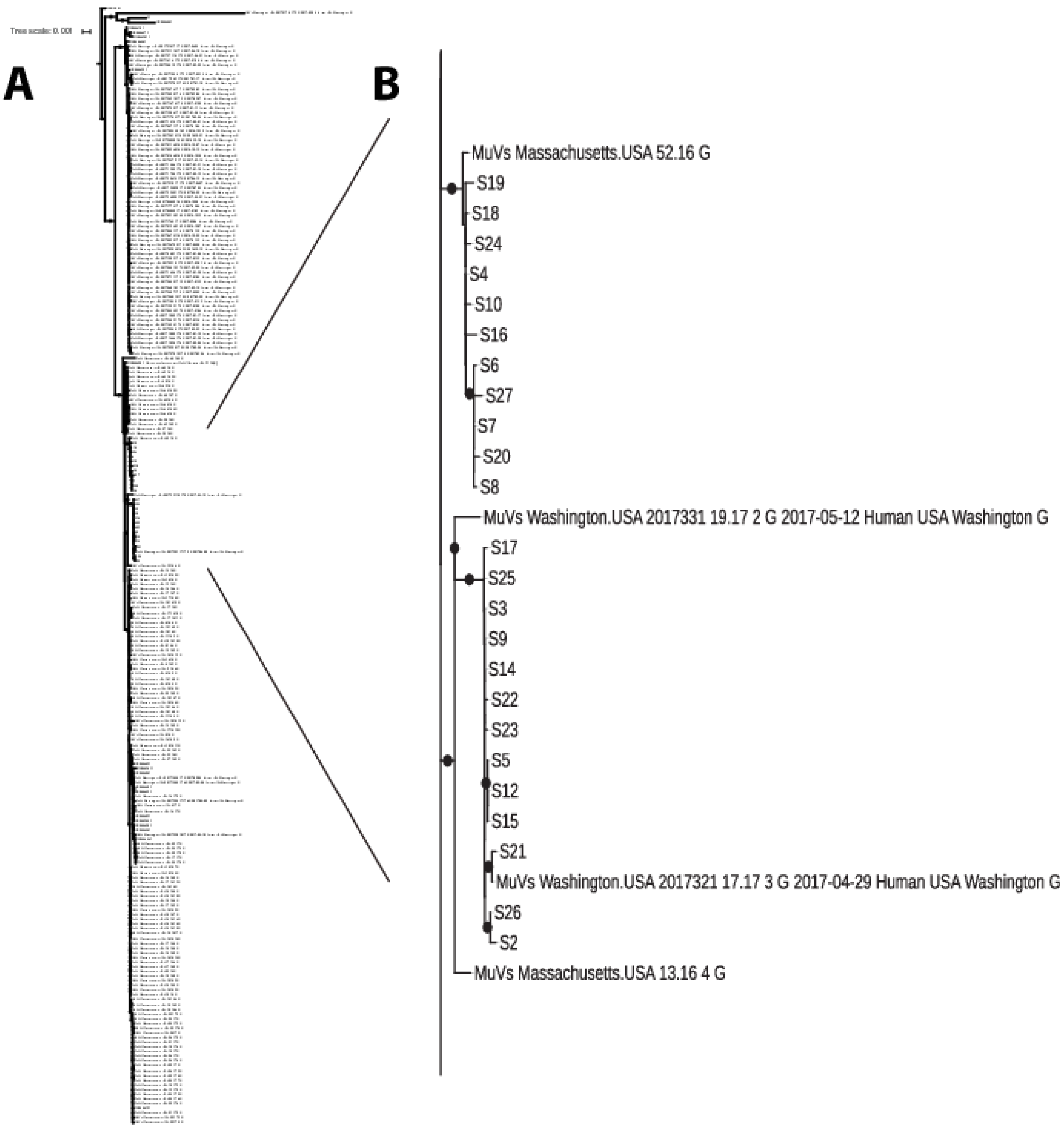

Supplementary Figure 1. (A) Maximum Likelihood tree of 211 mumps Genotype G samples from US in 2016 and 2017 and 25 Ontario 2017 isolates. The tree is rooted on JX287389.1, an ancestral strain from New York in 2012. (B) Enlargement of the Ontario outbreak clades, showing closely related Massachusetts and Washington strains. Nodes with ultrafast bootstrap support values >90 are indicated with black circles.
